# Dynamic analysis of the fractional distributed delay models

**DOI:** 10.1038/s41598-026-52327-8

**Published:** 2026-05-26

**Authors:** H. A. A. El-Saka, D. El. A. El-Sherbeny, A. M. A. El-Sayed

**Affiliations:** 1 Mathematics Department, Faculty of Science, Damietta University, New Damietta, 34517 Egypt; 2 Faculty of Artificial Intelligence and Information, Horus University, New Damietta, Egypt; 3 Faculty of Science, Alexandria University, Alexandria, 21526 Egypt

**Keywords:** Distributed delay models, Incommensurate fractional order systems, Stability analysis, Numerical solutions, Engineering, Mathematics and computing, Physics

## Abstract

In this paper, we analyze the stability of the fractional distributed delay models. We use the linear chain trick to convert these models into an incommensurate fractional order systems. We get the stability regions by studying the characteristic equation around equilibrium points. We investigate how the fractional order $\alpha _{1}$, $\rho$ and *a* affect the stability of the models. We study the fractional order delay logistic equation and compare the influence of the distributed delay on the stability regions. Numerical simulations are exhibited to confirm the analytical results.

## Introduction

Fractional calculus (FC) is one of the most important fields of mathematics. It’s an effective tool for explaining a wide range of scientific and engineering phenomena. Fractional order differential equations proposed natural models to study real-world problems, such as signal processing, biological mathematics, control processing, viscoelastic system^1–4^. The differential equation is known as a delayed differential equation (DDE) if the state variable appears with a delayed argument. Differential equation models with delay have become common in many technical and scientific fields in recent decades, such as biology, physics, neutral networks, and epidemiology^5–11^. The fractional delay differential equation (FDDE) has been applied for several years in many fields^3,4,12–15^. Fractional order delay differential equations can be better applied in biological, economic and social systems where memory effects play a role than in integer order equations.

Delay differential equations with distributed delay can be represented in general as

$$\begin{aligned} \dot{x}=F(t,x(t),\int _{0}^{\infty }\mu (\tau )x(t-\tau )). \end{aligned}$$

The distributed fractional-order nonlinear dynamical systems were first studied by Caputo in 1969^16^. Distributed delay differential equations are particularly attractive for modeling evaluation due to their long delays and mathematical tractability^5^. Recently, the application of distributed delay models has been used in a number of fields. For example, in biology, Compbell et al.^17^ studied how distributed delays arise in biological models and reviewed the literature on such models. Cushing^18^ discussed the stability and bifurcation analysis of some specific biological models. Some linear stability results for general distribution have been obtained by N. MacDonald^19,20^. In neural network, Liu et al.^21^ investigated the dynamical behaviors of a fractional-order neural network with leakage, discrete and distributed delays. Zhou et al.^22^ discussed stability and Hopf bifurcation analysis on a two neuron network with both discrete and distributed delays. Karaoğlu et al.^23^ investigated local stability and Hopf bifurcation in a two—neuron network model with multiple discrete and distributed delays. In ecological model, Yu et al.^24^ studied the dynamic complexities of an ecological model with impulsive control strategy and distributed time delay. AV. Paparao^25^ investigated the stability analysis of three species ecological model with distributed time delay. In control systems, Riccati equations with distributed delay are necessary for developing robust control systems and improving their performance. El-Sayed et al.^26^ investigated the dynamic properties of Riccati differential equation with distributed delay. In neurology, Karmeshu et al.^27^ analyzed a neuronal model with distributed delay. Liao et al.^28^ investigated a general two neuron model with distributed delays and a weak kernel. Chen et al.^29^ explored the extended strictly positive realness of fractional order systems with distributed delays (FOSDDs) and the application to robust control. Kiskinov et al.^30^ studied a general class of retarded linear systems with distributed delays and variable-order fractional derivatives of Caputo type. Chen et al.^31^ explored an order-dependent stability condition for nominal FOSDDs which is obtained through utilizing the small gain theorem. Popivanov et al.^32^ introduced an appropriate definition of the notion of a Lagrange (formal) adjoint system for homogeneous systems with incommensurate-order derivatives in Caputo’s sense and distributed delays.

The objective of this paper is to investigate the dynamic analysis of the fractional distributed delay models (20) and (24). In Section "Fractional order delay logistic equation", we obtained the stability analysis of the fractional order delay logistic equation by using the critical curve method. In Section "Fractional distributed delay models", by using the linear chain trick, we transform these models into an incommensurate fractional order systems. Section "Numerical simulations" contains the numerical simulations.

## Fractional order delay logistic equation

### Definition 2.1

^33^ The Riemann-Liouville fractional integral operator of order $\alpha \in R^{+}$ of the function *f*(*t*), $t \ge a$, is defined by

$$\begin{aligned} I^{\alpha }_{a}f(t)= \frac{1}{\Gamma {(\alpha )}} \int _{a}^{t} (t-\tau )^{\alpha -1} f(\tau )\text {d}\tau , \end{aligned}$$

where $\Gamma (.)$ is the Gamma function. When $a=0$, we set $I^{\alpha }_{0}f(t)= I^{\alpha }f(t)$ for simplicity.

### Definition 2.2

^33^ The Caputo fractional derivative of order $\alpha >0$ of *f*(*t*), $t\ge a$, is defined by

$$\begin{aligned} D^{\alpha }_{a}f(t)= {I^{n-\alpha }_{a}} D^{n} f(t), \end{aligned}$$

where $D=\textstyle \frac{d}{dt}$, and $n-1 < \alpha \le n$, $n\in N$. For simplicity, we set $D^{\alpha }_{0}f(t)= D^{\alpha }f(t)$ when $a=0$.

For the main properties of fractional calculus, see^33–38^.

In this section, we first consider the work of El-Saka et al.^12^, who studied the stability analysis and Hopf bifurcation of a fractional-order logistic equation with two different delays $\tau _{1}$, $\tau _{2}$ $>0$

1 $$\begin{aligned} \begin{aligned} D^{\alpha }y(t)&=\rho y(t-\tau _{1})\left( 1-y(t-\tau _{2})\right) , \quad t>0,\rho >0, \\ y(t)&=\phi (t), \quad -\tau \le t \le 0, \end{aligned} \end{aligned}$$

where $D^{\alpha }$ is a Caputo fractional derivative of order $0< \alpha \le 1$, the initial condition $\phi (t)$ is continuous on $[-\tau ,0]$ and $\tau =max\{\tau _{1},\tau _{2}\}$.

By using the results in^12^, we proposed the fractional-order delay logistic equation

2 $$\begin{aligned} D^{\alpha }y(t) =\rho y(t)- \rho y^{2}(t-r), \quad r,\rho >0, t\in (0, T], \end{aligned}$$

3 $$\begin{aligned} y(t)= \phi (t), \quad t \leqslant 0. \end{aligned}$$

The equilibrium points of Eq. (2) satisfy the equation

4 $$\begin{aligned} \rho y^{*}(1-y^{*})=0, \end{aligned}$$

which yields

$$\begin{aligned} y^{*}_{1}=0,\quad y^{*}_{2}=1. \end{aligned}$$

We will investigate the stability of Eq. (2) by using the critical curve method^8^ and compare these results with the finding we will present in the next sections.

The characteristic equation associated with the fractional order logistic equation with time delay Eq. (2) can be written as

5 $$\begin{aligned} \lambda ^{\alpha }+\rho \left( 2y^{*} e^{-\lambda r}-1\right) =0. \end{aligned}$$

An equilibrium point $y^{*}$ is asymptotically stable if all the roots $\lambda _{i}$ of the characteristic Eq. (5) satisfy

6 $$\begin{aligned} Re(\lambda _{i})<0. \end{aligned}$$

### Theorem 2.1

*The equilibrium point* $y^{*}_{1}=0$ *of fractional order delay logistic Eq.* (2) *is unstable for any* $r \ge 0$.

Let $r>0$ and $\lambda =w+iu$, $w,u \in R$. When the value of $\lambda$ crosses the imaginary axis at $\lambda =iu$ we can observe a change in stability, and the characteristic Eq. (5) becomes

7 $$\begin{aligned} (i u)^{\alpha }+\rho (2y^{*}e^{-iu r}-1)=0. \end{aligned}$$

Separating real and imaginary parts in (7), we have

8 $$\begin{aligned} u^{\alpha }cos\left( \textstyle \frac{\alpha \pi }{2}\right) -\rho =-2\rho y^{*} cos(ur), \end{aligned}$$

9 $$\begin{aligned} u^{\alpha }sin\left( \textstyle \frac{\alpha \pi }{2}\right) =2\rho y^{*} sin(ur). \end{aligned}$$

Squaring and adding Eqs. (8), (9), we obtain

10 $$\begin{aligned} u^{2\alpha }-2\rho u^{\alpha }cos\left( \textstyle \frac{\alpha \pi }{2}\right) +\rho ^{2}\left( 1-4y^{*\,2}\right) =0, \end{aligned}$$

the solution of Eq. (10) is

11 $$\begin{aligned} u^{\alpha }_{1,2}=\rho cos\left( \textstyle \frac{\alpha \pi }{2}\right) \pm \rho \sqrt{4y^{*}-sin^{2}\left( \textstyle \frac{\alpha \pi }{2}\right) }. \end{aligned}$$

From Eq. (8), we obtain

12 $$\begin{aligned} r=\textstyle \frac{1}{u}\left( 2n\pi \pm arccos\left( \textstyle \frac{u^{\alpha }cos\left( \textstyle \frac{\alpha \pi }{2}\right) -\rho }{-2\rho y^{*}}\right) \right) ,\quad n=0,1,\ldots . \end{aligned}$$

The critical curves can be obtained by substituting from Eq. (10) in (10)

13 $$\begin{aligned} r_{1}(n)=\textstyle \frac{2n\pi + arccos\left( \textstyle \frac{\left( \rho cos\left( \textstyle \frac{\alpha \pi }{2}\right) \pm \rho \sqrt{4y^{*\,2}-sin^{2}\left( \textstyle \frac{\alpha \pi }{2}\right) }\right) cos\left( \textstyle \frac{\alpha \pi }{2}\right) -\rho }{-2\rho y^{*}}\right) }{\left( \rho cos\left( \textstyle \frac{\alpha \pi }{2}\right) \pm \rho \sqrt{4y^{*\,2}-sin^{2}\left( \textstyle \frac{\alpha \pi }{2}\right) }\right) ^{\textstyle \frac{1}{\alpha }}},\quad n=0,1,\ldots , \end{aligned}$$

14 $$\begin{aligned} r_{2}(n)=\textstyle \frac{2n\pi - arccos\left( \textstyle \frac{\left( \rho cos\left( \textstyle \frac{\alpha \pi }{2}\right) \pm \rho \sqrt{4y^{*\,2}-sin^{2}\left( \textstyle \frac{\alpha \pi }{2}\right) }\right) cos\left( \textstyle \frac{\alpha \pi }{2}\right) -\rho }{-2\rho y^{*}}\right) }{\left( \rho cos\left( \textstyle \frac{\alpha \pi }{2}\right) \pm \rho \sqrt{4y^{*\,2}-sin^{2}\left( \textstyle \frac{\alpha \pi }{2}\right) }\right) ^{\textstyle \frac{1}{\alpha }}},\quad n=1,2,\ldots . \end{aligned}$$

### Theorem 2.2

*The equilibrium point* $y_{2}^{*}=1$ *of Eq.* (2) *has only one stability region located between* $r=0$ *and the closest critical curve*

15 $$\begin{aligned} r_{1}(0)=\textstyle \frac{arccos\left( \textstyle \frac{\left( \rho cos\left( \textstyle \frac{\alpha \pi }{2}\right) \pm \rho \sqrt{4-sin^{2}\left( \textstyle \frac{\alpha \pi }{2}\right) }\right) cos\left( \textstyle \frac{\alpha \pi }{2}\right) -\rho }{-2\rho }\right) }{\left( \rho cos\left( \textstyle \frac{\alpha \pi }{2}\right) \pm \rho \sqrt{4-sin^{2}\left( \textstyle \frac{\alpha \pi }{2}\right) }\right) ^{\textstyle \frac{1}{\alpha }}}, \end{aligned}$$

*and undergoes Hopf bifurcation at this value*.

### Proof

Differentiating the characteristic Eq. (5) with respect to *r* ($r>0$), we get

$$\begin{aligned} \alpha \lambda ^{\alpha -1}\textstyle \frac{d\lambda }{dr}-2\rho y^{*} r e^{-\lambda r}\textstyle \frac{d\lambda }{dr}-2\rho y^{*}\lambda e^{-\lambda r}=0, \end{aligned}$$

$$\begin{aligned} \textstyle \frac{d\lambda }{dr}=\textstyle \frac{\lambda \rho - \lambda ^{\alpha +1}}{\alpha \lambda ^{\alpha -1}-r\rho +r \lambda ^{\alpha }} \end{aligned}$$,

$$\begin{aligned} \textstyle \frac{d\lambda }{dr}\mid _{w=0}=\textstyle \frac{(iu) \rho - (iu)^{\alpha +1}}{\alpha (iu)^{\alpha -1}-r\rho +r (iu)^{\alpha }}. \end{aligned}$$

On critical curves (13) and (14)

16 $$\begin{aligned} Re\left( \textstyle \frac{d\lambda }{dr}\right) \mid _{\lambda =iu}=\textstyle \frac{z_{1}z_{3}+z_{2}z_{4}}{z^{2}_{3}+z^{2}_{4}}, \end{aligned}$$

where

$$\begin{aligned} z_{1}=u\rho cos\left( \textstyle \frac{\alpha \pi }{2}\right) -u^{\alpha +1}cos\left( \textstyle \frac{(\alpha +1)\pi }{2}\right) , \end{aligned}$$

$$\begin{aligned} z_{2}=u \rho sin\left( \textstyle \frac{\alpha \pi }{2}\right) - u^{\alpha +1}sin\left( \textstyle \frac{(\alpha +1)\pi }{2}\right) , \end{aligned}$$

$$\begin{aligned} z_{3}=\alpha u^{\alpha -1}cos\left( \textstyle \frac{(\alpha -1)\pi }{2}\right) +r u^{\alpha }cos\left( \textstyle \frac{\alpha \pi }{2}\right) -\rho r, \end{aligned}$$

and

$$\begin{aligned} z_{4}=\alpha u^{\alpha -1}sin\left( \textstyle \frac{(\alpha -1)\pi }{2}\right) +r u^{\alpha }sin\left( \textstyle \frac{\alpha \pi }{2}\right) . \end{aligned}$$

We have

$$\begin{aligned} z_{1}z_{3}+z_{2}z_{4}=r \rho u^{\alpha +1}\left( 1-sin\left( \textstyle \frac{\alpha \pi }{2}\right) \right) +\alpha u^{2\alpha }- ru\rho ^{2} cos\left( \textstyle \frac{\alpha \pi }{2}\right) . \end{aligned}$$

Then

$$\begin{aligned} Re\left( \textstyle \frac{d\lambda }{dr}\right) \mid _{\lambda =iu}>0. \end{aligned}$$

This implies that there does not exist any eigenvalue with negative real part across the critical curves (13) and (14). On the other hand, the equilibrium point $y^{*}_{2}=1$ is asymptotically stable for $r=0$. Therefore, there is only one stability region enclosed by $r=0$ and the critical curve $r_{1}(0)$, closest to it.

**Fig. 1:**
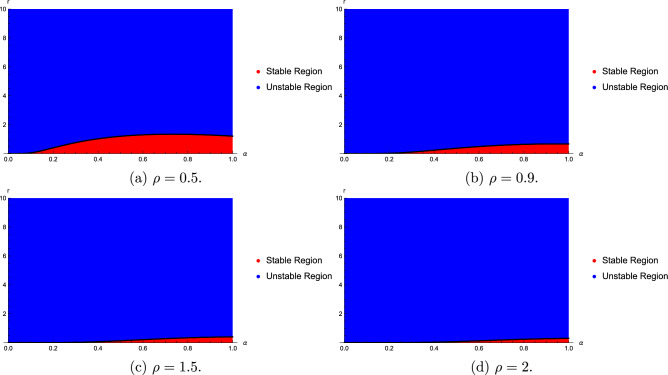
Stability regions with respect to $(\alpha ,r)$ when $\rho$ varies from 0.5 to 2.

**Fig. 2:**
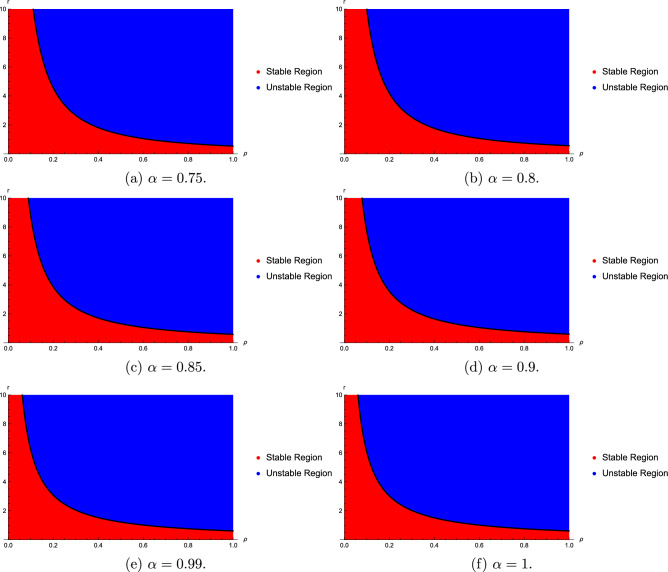
Stability regions with respect to $(\rho ,r)$ when $\alpha$ varies from 0.75 to 1.

We find that the stability regions are sensitive with the fractional order $\alpha$, $\rho$ and time delay *r*. Stability regions with respect to $\alpha$, $\rho$ and time delay are given in Figs. 1 and 2. Figures 1 and 2 show that as the values of $\rho$ and $\alpha$ become larger, the stability region becomes smaller.

## Fractional distributed delay models

### Preliminaries

#### Definition 3.1

^13^ The constant $(x^{*}_{1},x^{*}_{2},\dots ,x^{*}_{n})$ is an equilibrium point of the nonlinear fractional orders autonomous system

17 $$\begin{aligned} \begin{aligned} D^{\alpha _1}x_{1}(t)&= f_{1}\left( x_{1},x_{2},\dots ,x_{n}\right) ,\\ D^{\alpha _2}x_{2}(t)&=f_{2}\left( x_{1},x_{2},\dots ,x_{n}\right) , \\ \quad \vdots \\ D^{\alpha _n}x_{n}(t)&= f_{n}\left( x_{1},x_{2},\dots ,x_{n}\right) , \end{aligned} \end{aligned}$$

with the initial values

$$\begin{aligned} x_{1}(0)=x_{01}, x_{2}(0)=x_{02},\dots ,x_{n}(0)=x_{0n}, \end{aligned}$$

where all $\alpha _{i}$’s are rational numbers between 0 and 1, for $i=1,2,\dots ,n$, if and only if

$$\begin{aligned} f_{i}(x^{*}_{1},x^{*}_{2},\dots ,x^{*}_{n})=0, \quad i=1,2,\dots ,n. \end{aligned}$$

#### Theorem 3.1

^13, 39^ *Consider an incommensurate nonlinear fractional order system* (17). *Let M be the least common multiple (LCM) of the denominators* $u_{i}$’*s of* $\alpha _{i}$’*s where* $\alpha _{i}$ =$\textstyle \frac{v_{i}}{u_{i}}$, $(u_{i},v_{i})=1$ *(the greatest common divisor of* $u_{i}$ *and* $v_{i}$ *is 1),* $u_{i}$, $v_{i}$ $\in$ $Z^{+}$, $i=1,2,\dots ,n$. *Then the zero solution of system is asymptotically stable if and only if all roots* $\lambda 's$ *of the equation*

$$det \begin{pmatrix} \lambda ^{M\alpha _{1}}-a_{11} & -a_{12} & \cdots & -a_{1n}\\ -a_{21}& \lambda ^{M\alpha _{2}}-a_{22} & \cdots & -a_{2n}\\ \vdots & \vdots & \ddots & \vdots \\ -a_{n1} & -a_{n2} & \cdots & \lambda ^{M\alpha _{n}}-a_{nn} \end{pmatrix}$$*=0,*

$$\begin{aligned} a_{ij}=\frac{\partial f_{i}}{\partial x_{j}}|x^{eq}, \quad i,j=1,2,\dots ,n, \end{aligned}$$

*satisfy* $$\vert$$*arg(*$$\lambda$$*)*$$\vert$$ $$> \textstyle \frac{\pi }{2M}$$.

*This condition is equivalent to the following inequality*

18 $$\begin{aligned} \textstyle \frac{\pi }{2M}-\min \{\vert arg(\lambda )\vert \}<0, \end{aligned}$$

*which is called the instability measure for equilibrium points in fractional order systems (IMFOS).*

#### Proof

See^39^.

### Dynamic analysis of model I

We proposed the fractional distributed delay model as follows:

19 $$\begin{aligned} D^{\alpha _{1}}x(t)=\rho x(t)-\rho \left( \int _{0}^{t}e^{-a(t-s)}x(s)ds\right) ^{2}, \end{aligned}$$

$$\begin{aligned} x(0)=x_{0}, \end{aligned}$$

where $D^{\alpha _{1}}$ is a Caputo fractional derivative of order $0< \alpha _{1} < 1$, and $\rho ,a > 0$.

Let

$$\begin{aligned} y(t)=\int _{0}^{t}e^{-a(t-s)}x(s)ds, \end{aligned}$$

then, we get an incommensurate fractional order system as follows:

20 $$\begin{aligned} \begin{aligned} D^{\alpha _{1}}x(t)&=\rho x(t)-\rho y^{2}(t), \quad x(0)=x_{0}, \\ D^{\alpha _{2}}y(t)&=x(t)-a y(t), \quad y(0)=0, \end{aligned} \end{aligned}$$

where $$\alpha _{2}=1$$.

The system (20) has two equilibrium points

$$\begin{aligned} (x^{*}_{1},y^{*}_{1})=(0, 0), \quad (x^{*}_{2},y^{*}_{2})=(a^{2}, a). \end{aligned}$$

#### Theorem 3.2

*The equilibrium point* $(x^{*}_{1}, y^{*}_{1})=(0, 0)$ *of incommensurate system (*20) *is unstable for any* $\rho , a$ *and* $\alpha _{1}$.

#### Proof

According to Theorem 3.1, we find

$$\begin{aligned} det \begin{pmatrix} \lambda ^{M\alpha _{1}}-\rho & 0\\ -1& \lambda ^{M}+a \end{pmatrix}=0, \end{aligned}$$

21 $$\begin{aligned} \lambda ^{M\left( \alpha _{1}+1\right) }+a\lambda ^{M\alpha _{1}}-\rho \lambda ^{M}-a\rho =0, \end{aligned}$$

from Eq. (21), we obtain that the condition (18) is not verified,

where

$$\begin{aligned} min\{\vert arg(\lambda )\vert \}=0, \end{aligned}$$

hence IMFOS of the system is grater than zero.

#### Theorem 3.3

*The equilibrium point* $(x^{*}_{2}, y^{*}_{2})=(a^{2}, a)$ *of incommensurate system* (20) *is asymptotically stable if and only if all the root’s* $\lambda 's$ *of the equation*

22 $$\begin{aligned} \lambda ^{M\left( \alpha _{1}+1\right) }+a\lambda ^{M\alpha _{1}}-\rho \lambda ^{M}+a\rho =0, \end{aligned}$$

*satisfy* $$\vert$$*arg(*$$\lambda$$)$$\vert$$ $$> \textstyle \frac{\pi }{2M}$$.

#### Proof

The same as in Theorem 3.2.

We find that the stability regions are sensitive with the fractional order $\alpha _{1}$, $\rho$ and a. Stability regions for different $\rho$, $\alpha _{1}$ and *a* are given in Figs. 3, 4 and 5. As seen in Fig. 3, the stability region increases as $\rho$ decreases; however, when $\rho \le 1$, the entire region becomes stable, as shown in Fig. 3a. Figure 4 show that as the value of $\alpha _{1}$ become smaller, the stability region becomes smaller. Figure 5 shows that the stability domain increases as the values of *a* increase.

#### **Case 1: for different **$\rho$

**Fig. 3:**
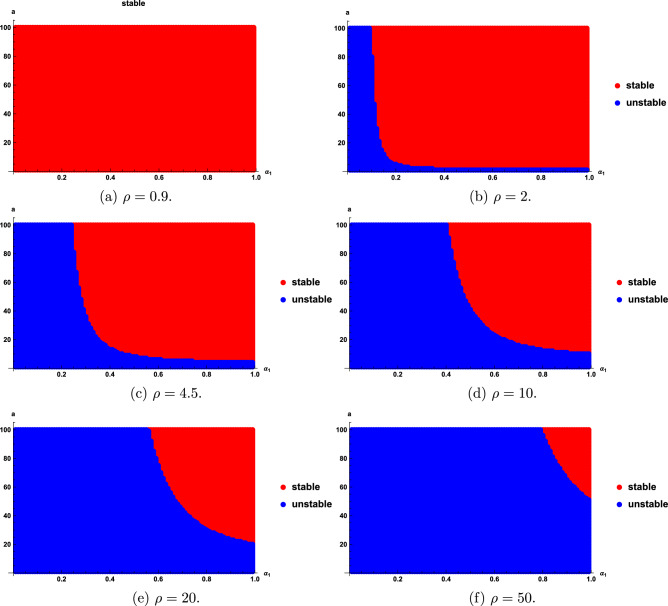
Stability regions of model (20) with respect to $(\alpha _{1},a)$ when $\rho$ varies from 0.9 to 50.

#### **Case 2: For different**$\alpha _{1}$

**Fig. 4:**
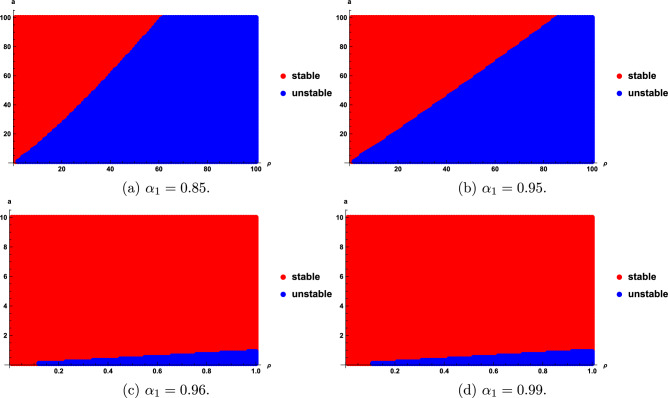
Stability regions of model (20) with respect to $(\rho ,a)$ when $\alpha _{1}$ varies from 0.85 to 0.99.

#### **Case 3: For different ***a*

**Fig. 5:**
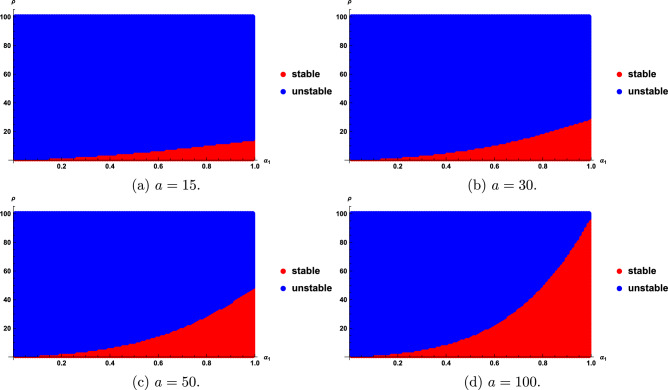
Stability regions of model (20) with respect to $(\alpha _{1},\rho )$ when *a* varies from 15 to 100.

Figs. 1, 2, 3, 4 and 5 show that the stability regions of model (20) are increased for the distributed delay model.

### Dynamic analysis of model II

We suggested the fractional distributed delay model as follows:

23 $$\begin{aligned} D^{\alpha _{1}}x(t)=\rho x(t)-\rho \int _{0}^{t}e^{-a(t-s)}x^{2}(s)ds, \end{aligned}$$

$$\begin{aligned} x(0)=x_{0}, \end{aligned}$$

where $D^{\alpha _{1}}$ is a Caputo fractional derivative of order $0< \alpha _{1} < 1$, and $a,\rho > 0$.

Let

$$\begin{aligned} y(t)=\int _{0}^{t}e^{-a(t-s)}x^{2}(s)ds, \end{aligned}$$

then, we obtain the feedback control as follows:

24 $$\begin{aligned} \begin{aligned} D^{\alpha _{1}}x(t)&=\rho x(t)-\rho y(t), \quad x(0)=x_{0}, \\ D^{\alpha _{2}}y(t)&=x^{2}(t)-a y(t), \quad y(0)=0, \end{aligned} \end{aligned}$$

where $$\alpha _{2}=1$$.

The model (24) have two equilibrium points

$$\begin{aligned} (x^{*}_{1},y^{*}_{1})=(0,0), \quad (x^{*}_{2},y^{*}_{2})=(a,a). \end{aligned}$$

#### Theorem 3.4

*The equilibrium point* $(x^{*}_{1}, y^{*}_{1})=(0, 0)$ *of incommensurate system* (24) *is unstable for any* $\rho , a$ *and* $\alpha _{1}$.

#### Theorem 3.5

*The equilibrium point* $(x^{*}_{2}, y^{*}_{2})=(a, a)$ *of incommensurate system* (24) *is asymptotically stable if and only if all the root’s* $\lambda 's$ *of the equation*

25 $$\begin{aligned} \lambda ^{M\left( \alpha _{1}+1\right) }+a\lambda ^{M\alpha _{1}}-\rho \lambda ^{M}+a\rho =0, \end{aligned}$$

*satisfy* $$\vert$$a*rg(*$$\lambda$$*)*$$\vert$$ $$> \textstyle \frac{\pi }{2M}$$.

For a detailed proof of Theorems 3.4 and 3.5, see Theorems 3.2 and 3.3.

We obtain that the stability regions are sensitive with the fractional order $\alpha _{1}$, *a*, and $\rho$. Stability regions with respect to $\alpha _{1}$, *a* and $\rho$ are shown in Figs. 6, 7, 8. Figure 6 show that the stability domain increases as the value of $\rho$ decreases. Figure 7 shows that the stability domain increases as the values of $\alpha _{1}$ increase. Figure 8 shows that the stability domain decreases as the values of *a* decrease.

#### **Case 1: For different**$\rho$

**Fig. 6:**
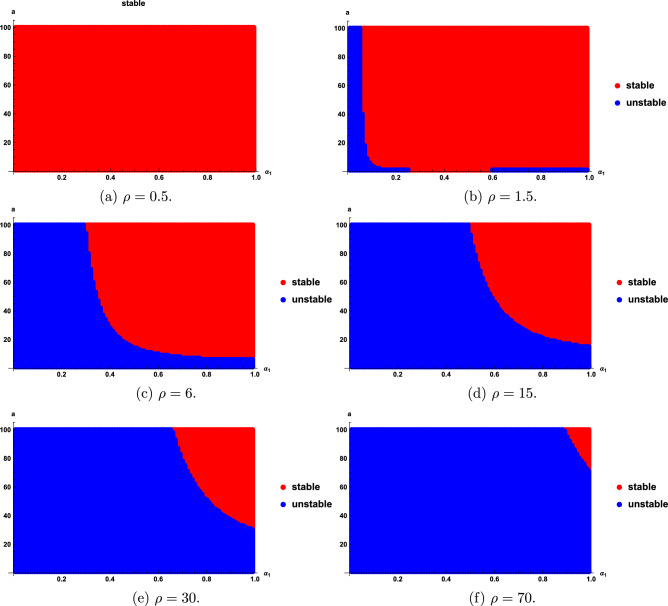
Stability regions of model (24) with respect to $(\alpha _{1},a)$ when $\rho$ varies from 0.5 to 70.

#### **Case 2: For different **$\alpha _{1}$

**Fig. 7:**
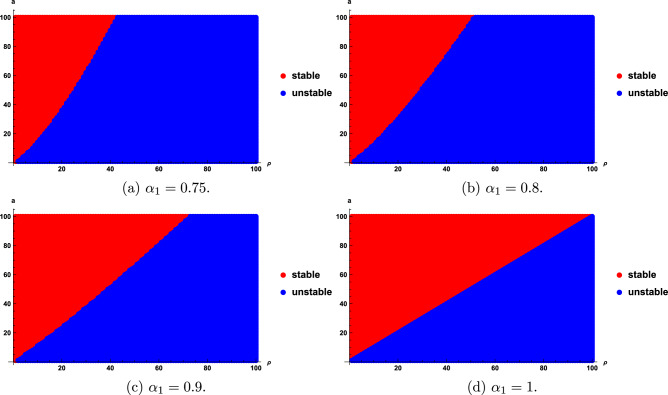
Stability regions of model (24) with respect to $(\rho ,a)$ when $\alpha _{1}$ varies from 0.75 to 1.

#### **Case 3: For different ***a*

**Fig. 8:**
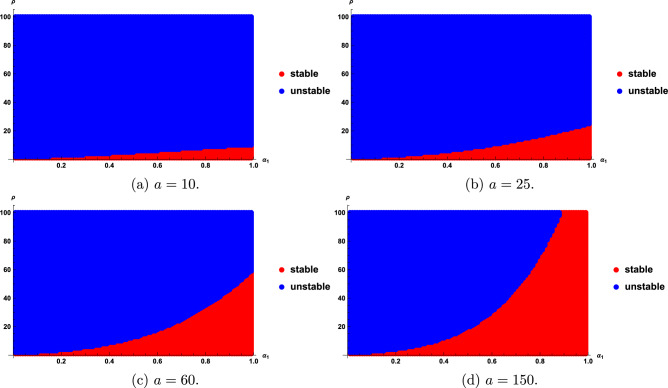
Stability regions of model (24) with respect to $(\alpha _{1},\rho )$ when *a* varies from 10 to 150.

From Figs. 1a-c, 2b-2f, 6, 7 and 8, we find that the distributed delay model (24) improves the stability regions.

## Numerical simulations

An adams-type predictor-corrector has been proposed and studied further in^36,40–43^. In this section, we use an Adams type predictor corrector method for the numerical solution of fractional integral equation.

### Model I

#### Case 1: For different *a*

**Fig. 9:**
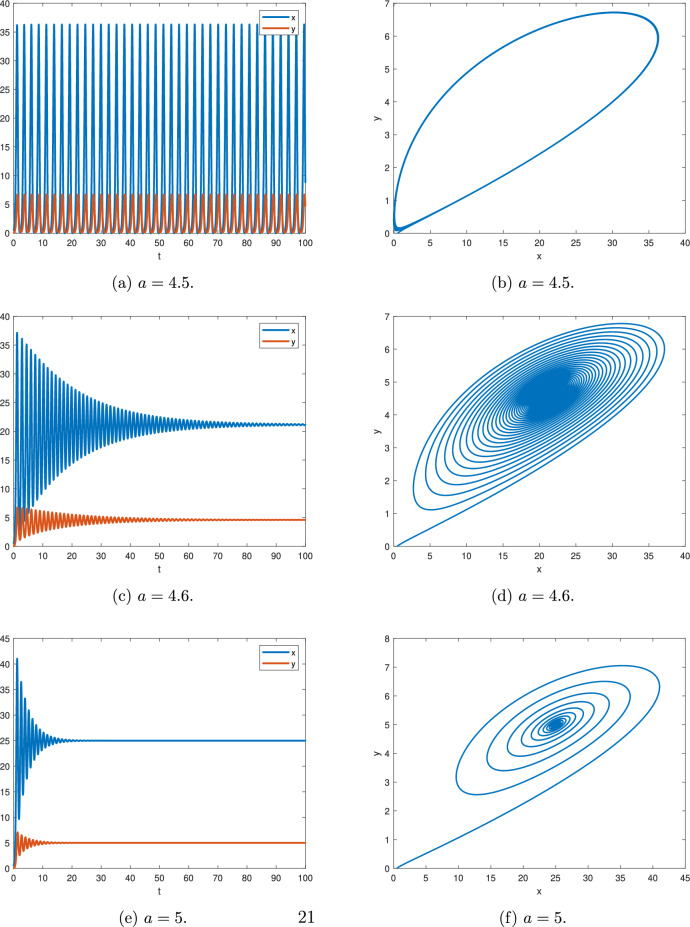
$$\alpha _{1}=0.97$$ and $$\rho =4.5.$$.

Figure 9 shows the numerical simulation for $\alpha _{1}=0.97$, $\rho =4.5$ and different *a*, where $\textstyle \frac{\Pi }{2M}=0.015708$.

Figure 9a,b, for $a=4.5$, according to Theorem 3.3, we have $min|arg(\lambda )|=0.0157111 \simeq \textstyle \frac{\Pi }{2M}$ and there is a periodic solution.

Figures 9c,d, for $a=4.6$, according to Theorem 3.3, we get $min|arg(\lambda )|=0.0158209 > \textstyle \frac{\Pi }{2M}$ and the equilibrium point $(a^{2},a)$ is asymptotically stable.

Figure 9e,f, for $a=5$, according to Theorem 3.3, we obtain $min|arg(\lambda )|=0.0162375 > \textstyle \frac{\Pi }{2M}$ and the equilibrium point $(a^{2},a)$ is asymptotically stable.

#### Case 2: For different $\rho$

**Fig. 10:**
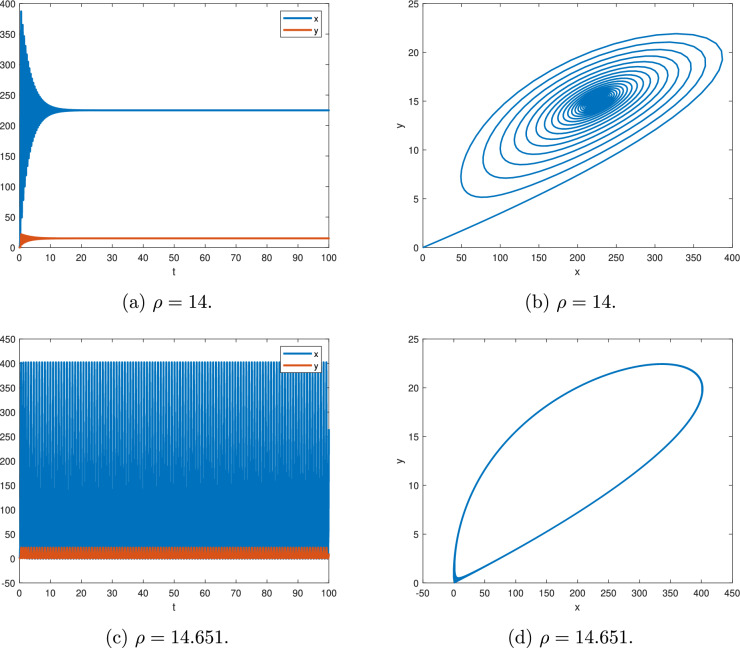
$$\alpha _{1}=0.98$$ and $$a=15$$.

Figure 10, for $$\alpha _{1}=0.98$$, $$a=15$$.

Figure 10a,c, for $\rho =14$, according to Theorem 3.3, we have $min|arg(\lambda )|=0.0159407 > \textstyle \frac{\Pi }{2M}$ and the equilibrium point $(a^{2},a)$ is asymptotically stable.

Figure 10c,d, for $\rho =14.651$, according to Theorem 3.3, we get $min|arg(\lambda )|=0.0157088 \simeq \textstyle \frac{\Pi }{2M}$ and there is a periodic solution.

### Model II

#### Case 1: For different *a*

**Fig. 11:**
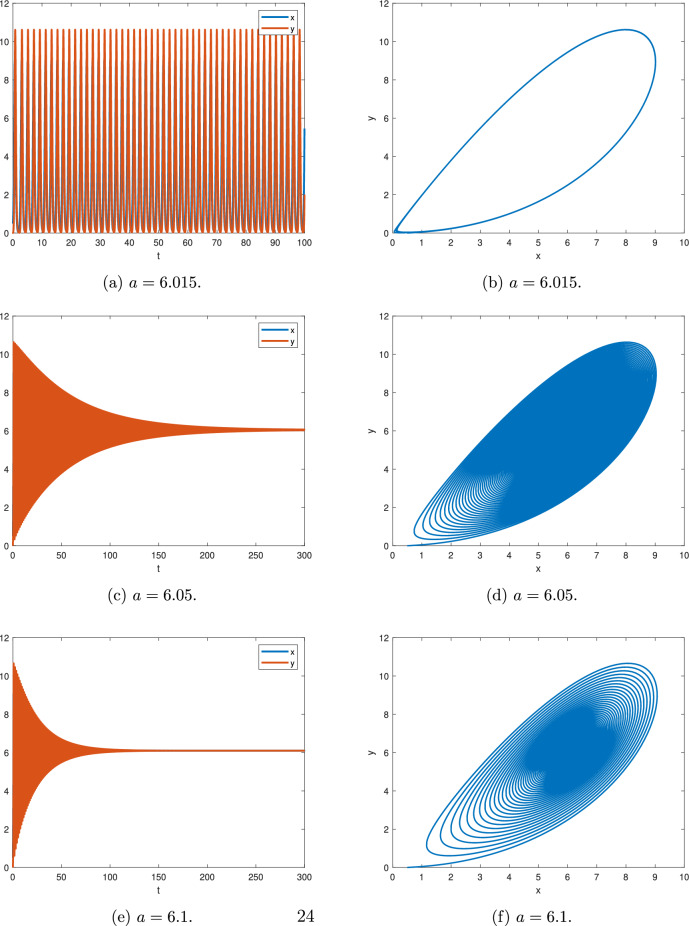
$$\alpha =0.99$$ and $$\rho =6$$.

Figure 11 shows the numerical simulation for $\alpha _{1}=0.99$, $\rho =6$ and different *a*, where $\textstyle \frac{\Pi }{2M}=0.015708$.

Figure 11a,b, for $a=6.015$, according to Theorem 3.5, we have $min|arg(\lambda )|=0.0157085 \simeq \textstyle \frac{\Pi }{2M}$ and there is a periodic solution.

Figure 11c,d, for $a=6.05$, according to Theorem 3.5, we get $min|arg(\lambda )|=0.0157375 > \textstyle \frac{\Pi }{2M}$ and the equilibrium point (*a*, *a*) is asymptotically stable.

Figure 11e,f, for $a=6.1$, according to Theorem 3.5, we obtain $min|arg(\lambda )|=0.0157787 > \textstyle \frac{\Pi }{2M}$ and the equilibrium point (*a*, *a*) is asymptotically stable.

#### Case 2: For different $\rho$

**Fig. 12:**
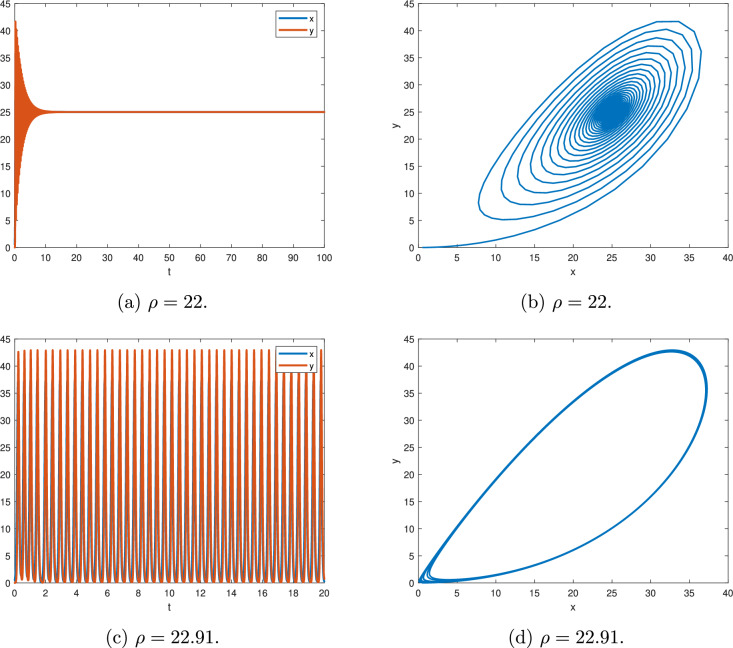
$$\alpha =0.95$$ and $$a=25$$.

Figure 12, for $$\alpha _{1}=0.95$$, $$a=25$$.

Figure 12a,b, for $\rho =22$, according to Theorem 3.5, we have $min|arg(\lambda )|=0.0159264> \textstyle \frac{\Pi }{2M}$ and the equilibrium point (*a*, *a*) is asymptotically stable.

Figure 12c,d, for $\rho =22.91$, according to Theorem 3.5, we get $min|arg(\lambda )|=0.0157135 \simeq \textstyle \frac{\Pi }{2M}$ and there is a periodic solution.

## Conclusions

In this paper, we studied the stability analysis of the fractional distributed delay models. The linear chain method transformed these models into an incommensurate fractional order systems with $0<\alpha _{1}<1$ and $\alpha _{2}=1$. We identified two equilibrium points $(x^{*}_{1},y^{*}_{1})=(0, 0)$, $(x^{*}_{2},y^{*}_{2})=(a^{2}, a)$ for model I and $(x^{*}_{1},y^{*}_{1})=(0,0)$, $(x^{*}_{2},y^{*}_{2})=(a,a)$ for model II. Theorems 3.2 and 3.4 indicate that the point $(x^{*}_{1},y^{*}_{1})$ of models I and II is unstable for any $\rho , a$ and $\alpha _{1}$. We determined the stability regions of the equilibrium point $(x^{*}_{2},y^{*}_{2})$ for models I and II by using the sufficient condition in Theorems 3.3 and 3.5. Furthermore, the stability regions are shown to be sensitive to $\rho$ (see Figs. 3 and 6), fractional order $\alpha _{1}$ (see Figs. 4 and 7), and *a* (see Figs. 5 and 8). We also proposed the fractional delay logistic equation and compared how the stability region of the distributed delay models (20) and (24) is better than the standard delay model (2). Numerical simulations illustrate the results. As a future research direction, the proposed approach will be extended to more complex nonlinear biological models involving time delays and incommensurate order.

## Data Availability

All data generated or analyzed during this study are included in this published article.
